# Contralateral breast cancer risk in patients with ductal carcinoma in situ and invasive breast cancer

**DOI:** 10.1038/s41523-020-00202-8

**Published:** 2020-11-03

**Authors:** Daniele Giardiello, Iris Kramer, Maartje J. Hooning, Michael Hauptmann, Esther H. Lips, Elinor Sawyer, Alastair M. Thompson, Linda de Munck, Sabine Siesling, Jelle Wesseling, Ewout W. Steyerberg, Marjanka K. Schmidt

**Affiliations:** 1 Division of Molecular Pathology, The Netherlands Cancer Institute, Amsterdam, the Netherlands; 2 Department of Biomedical Data Sciences, Leiden University Medical Center, Leiden, the Netherlands; 3 Department of Medical Oncology-Cancer Epidemiology, Erasmus MC Cancer Institute, Rotterdam, Netherlands; 4 Institute of Biostatistics and Registry Research, Brandenburg Medical School, Neuruppin, Germany; 5 Department of Epidemiology and Biostatistics, The Netherlands Cancer Institute, Amsterdam, the Netherlands; 6 School of Cancer & Pharmaceutical Sciences, Kings College London, London, UK; 7 Department of Surgery, Dan L Duncan Comprehensive Cancer Center, Baylor College of Medicine, Houston, USA; 8 Department of Research and Development, Netherlands Comprehensive Cancer Organisation, Utrecht, the Netherlands; 9 Department of Health Technology and Services Research, Technical Medical Centre, University of Twente, Enschede, the Netherlands; 10 Department of Pathology, The Netherlands Cancer Institute, Amsterdam, the Netherlands; 11 Department of Public Health, Erasmus MC, Rotterdam, the Netherlands

**Keywords:** Breast cancer, Cancer epidemiology, Risk factors

## Abstract

We aimed to assess contralateral breast cancer (CBC) risk in patients with ductal carcinoma in situ (DCIS) compared with invasive breast cancer (BC). Women diagnosed with DCIS (*N* = 28,003) or stage I–III BC (N = 275,836) between 1989 and 2017 were identified from the nationwide Netherlands Cancer Registry. Cumulative incidences were estimated, accounting for competing risks, and hazard ratios (HRs) for metachronous invasive CBC. To evaluate effects of adjuvant systemic therapy and screening, separate analyses were performed for stage I BC without adjuvant systemic therapy and by mode of first BC detection. Multivariable models including clinico-pathological and treatment data were created to assess CBC risk prediction performance in DCIS patients. The 10-year cumulative incidence of invasive CBC was 4.8% for DCIS patients (CBC = 1334). Invasive CBC risk was higher in DCIS patients compared with invasive BC overall (HR = 1.10, 95% confidence interval (CI) = 1.04–1.17), and lower compared with stage I BC without adjuvant systemic therapy (HR = 0.87; 95% CI = 0.82–0.92). In patients diagnosed ≥2011, the HR for invasive CBC was 1.38 (95% CI = 1.35–1.68) after screen-detected DCIS compared with screen-detected invasive BC, and was 2.14 (95% CI = 1.46–3.13) when not screen-detected. The C-index was 0.52 (95% CI = 0.50–0.54) for invasive CBC prediction in DCIS patients. In conclusion, CBC risks are low overall. DCIS patients had a slightly higher risk of invasive CBC compared with invasive BC, likely explained by the risk-reducing effect of (neo)adjuvant systemic therapy among BC patients. For support of clinical decision making more information is needed to differentiate CBC risks among DCIS patients.

## Introduction

Contralateral breast cancer (CBC) is the most frequent second cancer reported after first invasive breast cancer (BC)^1–3^. The cumulative incidence of invasive CBC for women following invasive BC is ~0.4% per year^4–6^. Several studies have shown a decrease in CBC incidence as a result of (neo)adjuvant systemic therapies^6–8^.

Ductal carcinoma in situ (DCIS) is a potential precursor of invasive BC. The incidence of DCIS has increased substantially with widespread introduction of population-based mammography screening including digital mammography and represents 10–25% of all BC patients^9–11^. As DCIS has an excellent prognosis with a disease-specific survival of >98% at 10 years^12–14^, a large group of women is at risk of developing CBC.

The risk of invasive CBC for DCIS patients has not been widely investigated, but the annual risk is estimated between 0.4 and 0.6%^11,13,15,16^. Moreover, it is unclear if the risk of CBC is comparable between patients diagnosed with invasive BC and patients with DCIS. One study in the United States, using data from the Surveillance, Epidemiology, and End Results (SEER) database, found a similar relative CBC risk for DCIS patients compared to patients with invasive BC^17^. On the other hand, an indirect assessment between DCIS patients and invasive BC patients has been provided by a CBC risk prediction model developed and validated in the USA, showing a higher relative CBC risk for DCIS compared with invasive BC (relative risk: 1.60, 95% confidence interval (CI) = 1.42–1.93)^18,19^. The reason for a potential higher CBC risk for DCIS patients is still unclear, but might relate to the risk-reducing effect of adjuvant systemic therapy among invasive BC patients^6,20,21^. In general, relatively few DCIS patients receive adjuvant systemic therapy. In addition, CBC risks may also differ based on the mode of detection of the first BC. Previous research showed that screen-detected invasive breast tumors have a better BC-specific survival than non-screened tumors and hence receive less adjuvant systemic treatment^22^.

The aim of this study was to assess the risk of developing invasive CBC in DCIS patients in direct comparison with patients diagnosed with invasive BC using a large population-based cohort of Dutch BC patients, taking age, mode of first BC detection, and (neo)adjuvant systemic therapy into account. In addition, we evaluated the CBC risk prediction performance in patients diagnosed with DCIS.

## Results

### Patient characteristics

The cohort comprised 28,003 DCIS patients (CBC = 1334) and 275,836 patients with invasive BC (CBC = 12,821), including 86,481 patients with stage I BC not receiving adjuvant systemic therapy; i.e., no chemotherapy, endocrine therapy, nor trastuzumab (Table 1). The percentage of patients diagnosed with DCIS, of all BC patients diagnosed in the Netherlands, was 5.7% in the implementation phase of the mammography screening program (1989–1998) and 10.5% in the period of full national coverage (1999–2017). Median follow-up was 11.4 years.

**Table 1:** Patient-, tumor-, and treatment characteristics of women diagnosed with ductal carcinoma in situ or invasive breast cancer.

	DCIS		All invasive BC		Stage I BC without adjuvant systemic therapy^a^	
	*N*	%	*N*	%	*N*	%
Characteristics	28,003	9.2	275,836	90.8	86,481	31.4
Diagnosis, year						
Median (range)	2009 (1989–2017)		2004 (1989–2017)		2004 (1989–2017)	
Age, years						
Median (range)	59 (21–95)		59 (18–102)		61 (18–99)	
*TNM stage*						
0	28,003	100.0	–	–	–	–
I	–	–	120,952	43.8	86,481	100.0
II	–	–	124,883	45.3	–	–
III	–	–	30,001	10.9	–	–
*Tumor grade*						
I (well differentiated)	3729	16.1	44,690	20.9	27,566	41.9
II (moderately differentiated)	7864	33.8	95,251	44.6	28,159	42.8
III (poorly/undifferentiated)	11,639	50.1	73,581	34.5	10,036	15.3
Missing	4771	–	62,314	–	20,720	–
*ER status*						
Positive	–	–	133,761	82.7	41,883	90.1
Negative	–	–	28,075	17.3	4598	9.9
Missing	28,003	–	114,000	–	40,000	–
*HER2 status*						
Positive	–	–	19,708	14.3	2324	6.1
Negative	–	–	118,409	85.7	35,616	93.9
Missing	28,003	–	137,719	–	48,541	–
*PR status*						
Positive	–	–	106,786	67.5	33,862	74.8
Negative	–	–	51,437	32.5	11,404	25.2
Missing	28,003	–	117,613	–	41,215	–
*(Neo)adjuvant chemotherapy*						
Yes	17	0.1	91,844	33.3	–	–
No	27,986	99.9	183,992	66.7	86,481	100.0
*(Neo)adjuvant endocrine therapy*						
Yes	102	0.4	119,394	43.3	–	–
No	27,901	99.6	156,442	56.7	86,481	100.0
*(Neo)adjuvant trastuzumab*						
Yes	3	0.0	13,994	5.1	–	–
No	28,000	100.0	261,842	94.9	86,481	100.0
*Surgery to the breast*						
Breast conserving surgery	16,396	60.8	142,495	53.4	58,727	70.1
Mastectomy	10,571	39.2	124,530	46.6	25,023	29.9
Missing	1036	–	881	–	2731	–
*Radiation to the breast*						
Yes	13,128	46.9	182,226	66.1	59,354	70.1
No	14,875	53.1	93,610	33.9	27,127	31.4
*Follow-up, years*						
Median (IQR)	8.7 (8.5–8.8)		11.8 (11.7–11.8)		13.5 (13.4–13.6)	
*Cumulative incidence of invasive CBC, %*						
5-year (95% CI)	2.4 (2.2–2.6)		2.0 (2.0–2.1)		2.9 (2.8–3.0)	
10-year (95% CI)	4.8 (4.6–5.2)		4.0 (4.0–4.1)		5.6 (5.4–5.8)	
Number of invasive CBC	1334		12,821		5782	
*Cumulative incidence of death, %*						
5-year (95% CI)	3.8 (3.6–4.0)		15.0 (14.9–15.2)		7.8 (7.6–8.0)	
10-year (95% CI)	9.8 (9.4–10.2)		29.4 (29.2–29.6)		19.2 (18.9–19.5)	
Number of death	3340		91,797		23,899	
*Cumulative incidence of ipsilateral invasive BC %*						
5-year (95% CI)	1.6 (1.5–1.8)		0.1 (0.1–0.1)		0.2 (0.1–0.2)	
10-year (95% CI)	3.5 (3.3–3.8)		0.3 (0.2–0.3)		0.5 (0.4–0.6)	
Number of ipsilateral invasive BC	920		1471		897	
*Cumulative incidence of in situ CBC, %*						
5-year (95% CI)	1.0 (1.0–1.1)		0.4 (0.4–0.5)		0.6 (0.6–0.7)	
10-year (95% CI)	1.6 (1.5–1.8)		0.8 (0.7–0.8)		1.1 (1.0–1.2)	
Number of in situ CBC	427		2278		1026	

*DCIS* ductal carcinoma in situ, *BC* breast cancer, *ER* estrogen receptor, *PR* progesterone receptor, *HER2* human epidermal growth factor receptor 2, *IQR* inter-quartile range, *CBC* contralateral breast cancer, *CI* confidence interval.

^a^The “stage I BC without adjuvant systemic therapy” group is a subset of the “all invasive BC” group.

### CBC risk for patients diagnosed with DCIS and invasive BC

**Fig. 1:**
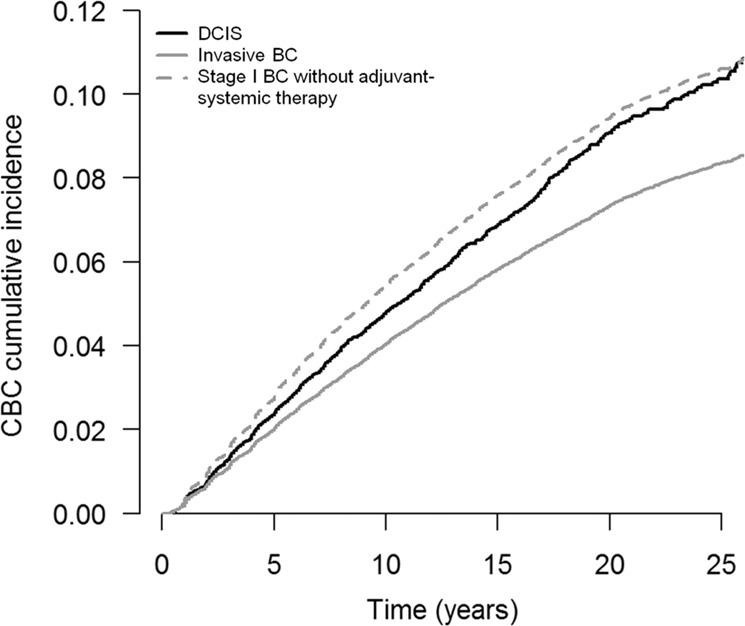
Cumulative incidences of invasive contralateral breast cancer (CBC) in patients diagnosed with ductal carcinoma in situ (DCIS), invasive breast cancer (BC) stage I–III, and stage I BC without (neo)adjuvant systemic therapy. The *x* axis represents the time since first BC diagnosis (in years) and the *y* axis the cumulative CBC incidence.

The 10-year cumulative incidence of invasive CBC was 4.8% (95% CI = 4.6–5.2%) for DCIS patients, 4.0% (95% CI = 4.0–4.1%) for all invasive BC patients, and 5.6% (95% CI = 5.4–5.8%) for patients with stage I BC not receiving adjuvant systemic therapy (Table 1, Fig. 1^23^). For comparison, the 10-year cumulative incidence of invasive CBC in patients diagnosed with stage I invasive BC treated with adjuvant systemic therapy was 2.9% (95% CI = 2.5–3.3%). Being diagnosed with DCIS was associated with an increased risk of invasive CBC compared with invasive BC overall (HR = 1.10, 95% CI = 1.04–1.17), and with a lower risk when compared with stage I BC without adjuvant systemic therapy (HR = 0.87, 95% CI = 0.82–0.92, Table 2). Similar results were observed when using competing risk regression (Table 2).

**Table 2:** Relative subsequent contralateral breast cancer risks (invasive and in situ) after diagnosis with ductal carcinoma in situ versus invasive breast cancer using Cox and competing risk regression.

		Cox regression		Competing risks regression	
Outcome(s)	Type of first BC	Unadjusted	Adjusted^a^	Unadjusted	Adjusted^a^
		HR (95% CI)	HR (95% CI)	HR^b^ (95% CI)	HR^b^ (95% CI)
Invasive CBC	DCIS vs invasive BC	1.08 (1.01–1.14)	1.10 (1.04–1.17)	1.22 (1.15–1.28)	1.20 (1.14–1.27)
	DCIS vs stage I BC without adjuvant systemic therapy	0.87 (0.82–0.92)	0.87 (0.82–0.92)	0.88 (0.83–0.94)	0.87 (0.82–0.93)
In situ CBC	DCIS vs invasive BC	1.92 (1.72–2.13)	1.84 (1.66–2.04)	2.12 (1.92–2.38)	1.98 (1.79–2.20)
	DCIS vs stage I BC without adjuvant systemic therapy	1.49 (1.33–1.67)	1.38 (1.22–1.55)	1.54 (1.37–1.72)	1.40 (1.25–1.58)

*HR* hazard ratio, *CI* confidence interval, *CBC* contralateral breast cancer, *BC* breast cancer, *DCIS* ductal carcinoma in situ.

^a^Hazard ratios adjusted by age and year at first diagnosis.

^b^Hazard ratios for the subdistribution hazards of the Fine and Gray model. Invasive CBC, in situ CBC, invasive ipsilateral BC, and death were taken into account as competing risks.

In sensitivity analyses using different time cutoffs for metachronous CBC, results were similar. The HR for invasive CBC developed at least six months after the first BC was 1.10 (95% CI = 1.04–1.17) for DCIS compared with invasive BC, and the HR was 1.09 (95% CI = 1.03–1.16) using a 12-month cutoff.

The cumulative incidence of in situ CBC, death, and invasive ipsilateral BC are shown in Supplementary Figs. 1–3^23^. The 10-year cumulative incidence of in situ CBC was 1.6% (95% CI = 1.5–1.8%) for DCIS patients, 0.8% (95% CI = 0.7–0.8%) for invasive BC patients, and 1.1% (95% CI = 1.0–1.2%) for patients with stage I BC without adjuvant systemic therapy (Table 1). The risk of death was lower in DCIS patients compared to invasive BC patients (HR = 0.47, 95% CI = 0.45–0.49, Supplementary Table 1).

### Results by age and screening (period)

**Fig. 2:**
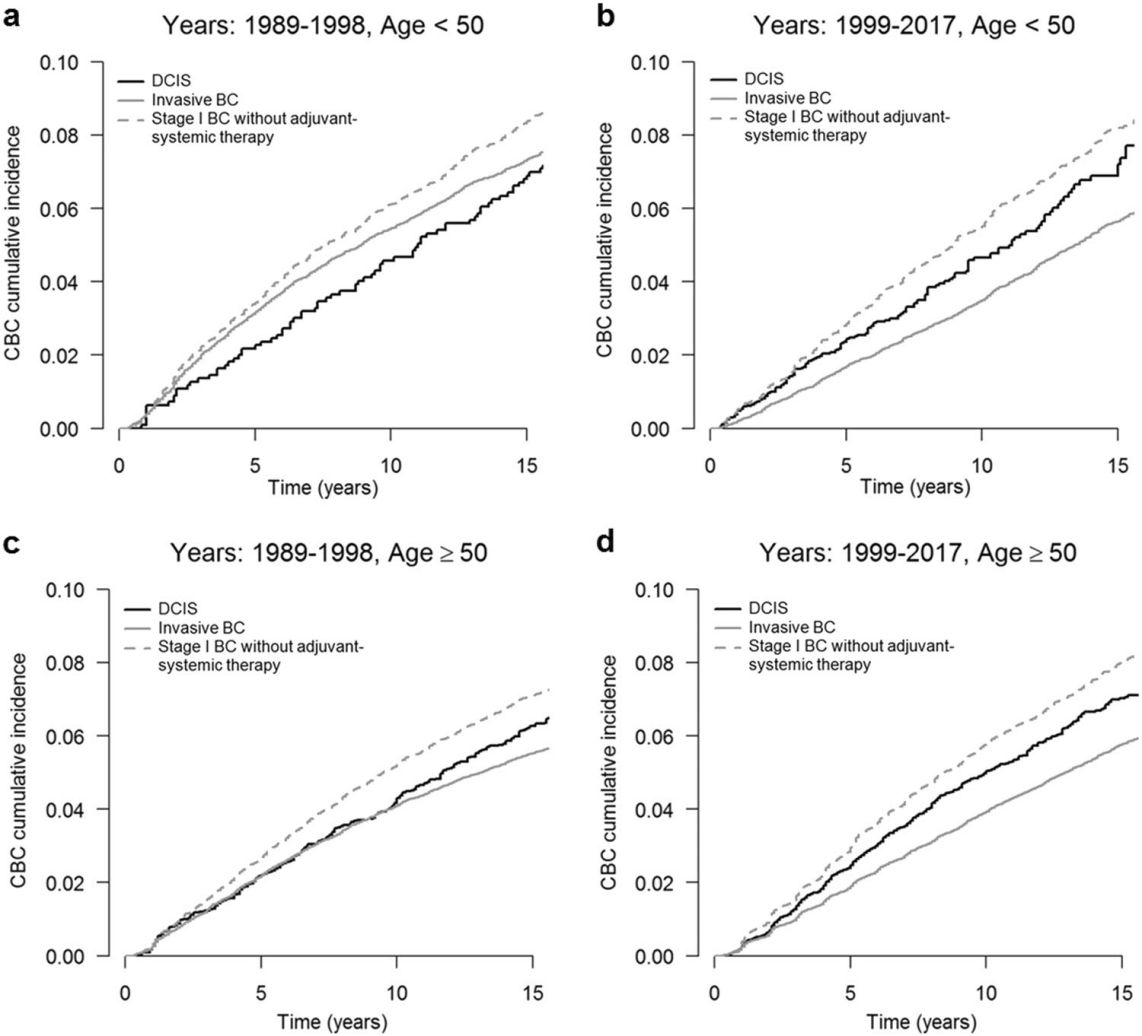
Cumulative incidences of invasive contralateral breast cancer (CBC) in patients diagnosed with ductal carcinoma in situ (DCIS), invasive breast cancer (BC) stage I–III, or stage I BC without (neo)adjuvant systemic therapy. **a** patients aged <50 years diagnosed between 1989 and 1998 (implementation phase Dutch mammography screening program); **b** patients aged <50 years diagnosed between 1999 and 2017 (full national coverage of the Dutch mammography screening program); **c** patients aged ≥50 years diagnosed between 1989 and 1998; **d** patients aged ≥50 years diagnosed between 1999 and 2017. The *x* axis represents the time since first BC diagnosis (in years) and the *y* axis the cumulative CBC incidence.

Among patients who had their first BC diagnosis during the implementation phase of the national screening program (1989–1998), the risk of invasive CBC was similar in DCIS patients compared with invasive BC patients (HR = 0.93, 95% CI = 0.85–1.03, Table 3, Fig. 2a–c^23^). In the period of full nationwide coverage of the screening program (1999–2017), the risk of invasive CBC was higher for DCIS patients than for invasive BC patients (HR = 1.19, 95% CI = 1.10–1.27, Table 3, Fig. 2b–d^23^). The risk of invasive CBC was lower in DCIS patients compared with patients with stage I BC not receiving adjuvant systemic therapy in both periods (1989–1998: HR = 0.90; 95% CI = 0.81–1.00, and 1999–2017: HR = 0.85, 95% CI: 0.79–0.91). The effects were similar stratified by age group (<50 and ≥50 years) (Table 3). The estimated 5- and 10-year cumulative incidences by age and period are shown in Supplementary Table 2.

**Table 3:** Relative risk of invasive contralateral breast cancer after ductal carcinoma in situ versus invasive breast cancer by period and age at first diagnosis using Cox and competing risks regression.

					Cox regression		Competing risks regression	
	Period	Type of first BC	*N*	CBC events	HR	95% CI	HR^a^	95% CI
*All*	1989–1998	DCIS vs invasive BC	81,105	6488	0.93	0.85–1.03	1.11	1.01–1.23
	1999–2017	DCIS vs invasive BC	222,734	7667	1.19	1.10–1.27	1.32	1.23–1.41
	1989–1998	DCIS vs stage I BC without systemic therapy	273,383	2696	0.90	0.81–1.00	0.93	0.85–1.04
	1999–2017	DCIS vs stage I BC without systemic therapy	59,098	3086	0.85	0.79–0.91	0.88	0.81–0.94
*Age <50 years at first diagnosis*^*b*^								
	1989–1998	DCIS vs invasive BC	22,084	2292	0.94	0.83–1.09	1.06	0.92–1.22
	1999–2017	DCIS vs invasive BC	53,570	1838	1.20	1.06–1.37	1.26	1.11–1.45
	1989–1998	DCIS vs stage I BC without systemic therapy	7192	870	0.90	0.78–1.04	0.89	0.78–1.04
	1999–2017	DCIS vs stage I BC without systemic therapy	8162	472	0.85	0.74–0.97	0.82	0.71–0.94
*Age ≥50 years at first diagnosis*^*b*^								
	1989–1998	DCIS vs invasive BC	59,021	4196	0.92	0.83–1.03	1.14	1.03–1.26
	1999–2017	DCIS vs invasive BC	169,164	5829	1.18	1.10–1.26	1.35	1.26–1.47
	1989–1998	DCIS vs stage I BC without systemic therapy	20,191	1826	0.89	0.80–1.00	0.96	0.86–1.08
	1999–2017	DCIS vs stage I BC without systemic therapy	50,936	2614	0.85	0.78–0.92	0.88	0.81–0.95

*HR* hazard ratio, *CI* confidence interval, *DCIS* ductal carcinoma in situ, *BC* breast cancer.

^a^Hazard ratios for the subdistribution hazards of the Fine and Gray model. Invasive CBC, in situ CBC, invasive ipsilateral BC, and death were taken into account as competing risks.

^b^Results were based on interaction analyses including the interaction term between age, period, and type of first BC (type of first BC+age+period+age×type of first BC+period×type of first BC).

In a subgroup of patients diagnosed during or after 2011, with information available on the mode of first BC detection, the HR of invasive CBC was 1.53 (95% CI = 1.29–1.82) for DCIS patients compared with invasive BC patients, and 0.86 (95% CI = 0.71–1.03) compared with patients with stage I BC without adjuvant systemic therapy (Table 4). Among all screen-detected first BCs, the HR of invasive CBC was 1.38 (95% CI = 1.35–1.68) for DCIS patients compared with invasive BC patients and 0.81 (95% CI = 0.66–1.00) compared with stage I BC without adjuvant systemic therapy (Table 4). When the first BC was not detected by screening, the HR of invasive CBC was 2.14 (95% CI = 1.46–3.13) for DCIS patients compared to invasive BC patients and 1.04 (95% CI = 0.68–1.59) compared with stage I BC without adjuvant systemic therapy (Table 4). The risk of death in patients with DCIS compared with invasive BC and stage I BC without adjuvant systemic therapy among screen-detected and not screen-detected is shown in Supplementary Table 3.

**Table 4:** Relative subsequent event risks after diagnosis with ductal carcinoma in situ versus invasive breast cancer by mode of first breast cancer detection for patients diagnosed between 2011 and 2017^a^.

		Overall		By mode of first BC detection^b^		
		Cox regression	Competing risks regression		Cox regression	Competing risks regression
Outcome	Type of first BC	HR (95% CI)^c^	HR^c,d^ (95% CI)		HR^c^ (95% CI)	HR^c,d^ (95% CI)
Invasive CBC	DCIS vs invasive BC (*n* = 62,533, events=763)	1.53 (1.29–1.82)	1.55 (1.30–1.85)	Screen-detected^e^	1.38 (1.35–1.68)	1.38 (1.13–1.69)
				Not screen-detected^e^	2.14 (1.46–3.13)	2.20 (1.50–3.22)
	DCIS vs stage I BC without systemic therapy (*n* = 27,288, events = 519)	0.86 (0.71–1.03)	0.86 (0.71–1.03)	Screen-detected^e^	0.81 (0.66–1.00)	0.81 (0.65–1.00)
				Not screen-detected^e^	1.04 (0.68–1.59)	1.05 (0.68–1.60)
In situ CBC	DCIS vs invasive BC (*n* = 62,533, events = 250)	1.99 (1.51–2.63)	2.00 (1.52–2.65)	Screen-detected^e^	1.75 (1.26–2.45)	1.75 (1.26–2.45)
				Not screen-detected^e^	3.41 (1.98–5.87)	3.46 (2.01–5.97)
	DCIS vs stage I BC without systemic therapy (*n* = 27,288, events = 146)	1.51 (1.08–2.10)	1.51 (1.08–2.10)	Screen-detected^e^	1.40 (0.96–2.06)	1.41 (0.96–2.06)
				Not screen-detected^e^	2.23 (1.14–4.39)	2.25 (1.15–4.41)

*BC* breast cancer, *HR* hazard ratio, *CI* confidence interval, *CBC* contralateral breast cancer, *DCIS* ductal carcinoma in situ.

^a^The analyses were performed in all patients diagnosed between 2011–2017, since from 2011 we had virtually complete information on the mode of first BC detection.

^b^Results were based on interaction analyses including the interaction term between mode of first BC detection and type of first BC (type of first BC+mode of first BC detection+mode of first BC detection×type of first BC).

^c^Adjusted for age at first BC diagnosis.

^d^Hazard ratios for the subdistribution hazards of the Fine and Gray model. Invasive CBC, in situ CBC, invasive ipsilateral BC, and death were taken into account as competing risks.

^e^Not screen-detected includes interval tumors, non-screen attendant, or screened outside the national program.

### Subtype-specific CBC risk

DCIS patients had a lower risk of stage IV CBC (HR = 0.45, 95% CI = 0.22–0.92), and higher risks of grade I invasive CBC (HR = 1.55, 95% CI = 1.31–1.84) and ER-positive invasive CBC (HR = 1.49, 95% CI = 1.33–1.66) compared with all invasive BC patients (Supplementary Table 4). Overall, the subtype-specific CBC risk in DCIS patients was comparable to patients with stage I BC not receiving adjuvant systemic therapy (Supplementary Table 4).

### Multivariable model

In the multivariable model, no strong predictors of CBC were identified in DCIS patients (Table 5). The C-index of the multivariable model of invasive CBC was 0.52 (standard deviation (SD = 0.01) for cause-specific Cox regression; when we considered all CBC (in situ and invasive) the C-index was 0.51 (SD = 0.01) (Table 5). When we performed the analyses in a subgroup of patients diagnosed during or after 2011, the C-index was 0.55 (SD = 0.01) without information on the mode of first BC detection, and 0.56 (SD = 0.01) with information available on the mode of first BC detection (data not shown).

**Table 5:** Relative risks of invasive and in situ contralateral breast cancer after diagnosis with ductal carcinoma in situ or invasive breast cancer using multivariable Cox and competing risk regression models.

Outcome	Invasive CBC				Invasive and in situ CBC			
	Cox regression		Competing risk regression		Cox regression		Competing risk regression	
	HR	95% CI	HR^a^	95% CI	HR	95% CI	HR^a^	95% CI
Age (years)	1.01^b^	0.93–1.10	0.78^c^	0.69–0.89	0.93^b^	0.87–1.00	0.71^c^	0.63–0.81
Tumor grade								
Moderately differentiated versus well differentiated	0.93	0.78–1.12	0.94	0.79–1.12	0.99	0.85–1.16	0.99	0.85–1.16
Poorly differentiated versus well differentiated	0.92	0.76–1.10	0.93	0.77–1.11	0.94	0.81–1.09	0.94	0.81–1.09
Surgery (Mastectomy versus BCS)	0.96	0.80–1.16	1.00	0.83–1.21	1.08	0.92–1.26	1.13	0.96–1.32
Radiotherapy to the breast (yes versus no)	1.11	0.94–1.32	1.12	0.94–1.33	1.12	0.97–1.30	1.14	0.98–1.32
Baseline failure-free probability at 10 years^d^	0.949		0.956^e^		0.932		0.943^e^	
C-index (SD)	0.520 (0.01)		0.515 (0.01)		0.513 (0.01)		0.526 (0.01)	

*CBC* contralateral breast cancer, *HR* hazard ratio, *CI* confidence interval, *BCS* breast conservative surgery, *SD* standard deviation.

^a^Hazard ratios for the subdistribution hazards of the Fine and Gray model.

^b^Parameterized per decade.

^c^Parameterized as a restricted cubic spline with three knots.

^d^The baseline failure-free probabilty function is calculated for baseline values of the predictors included in the multivariable models.

^e^Baseline failure-free probability function for the subdistribution hazard of the Fine and Gray model.

## Discussion

In this large population-based study, the 10-year cumulative incidence of invasive CBC was 4.8% for DCIS patients. The risk of developing invasive CBC was lower for DCIS patients compared with stage I BC patients not receiving adjuvant systemic therapy (HR = 0.87), but the risk was slightly higher compared with all invasive BC patients (HR = 1.10). A multivariable model, based on the clinical information currently available, was unable to differentiate risks of invasive CBC among DCIS patients.

The slightly higher invasive CBC risk in DCIS patients compared with all invasive BC patients may be explained by the risk-reducing effect of adjuvant systemic therapy among invasive BC patients^6,20,21^. In our previous study using NCR data^6^ we showed that adjuvant endocrine therapy, chemotherapy, and trastuzumab combined with chemotherapy were associated with overall 54%, 30%, and 43% risk reductions of CBC, respectively. In our study, a large group (57%) of patients with invasive BC received (neo)adjuvant systemic therapy. According to the Dutch guidelines, DCIS patients are not offered treatment with adjuvant systemic therapy^24^. The potential influence of adjuvant systemic therapy is supported by the CBC risk evaluation in patients diagnosed with stage I BC not receiving adjuvant systemic therapy, showing a higher CBC risk in such patients than in patients diagnosed with DCIS.

To our knowledge, only one previous study in the United States investigated the risk of CBC in patients with DCIS in direct comparison with patients diagnosed with invasive BC using SEER data^17^. They found a similar CBC risk (including in situ and invasive) for invasive ductal BC in comparison with DCIS, with a relative risk of 0.98 (95% CI = 0.90–1.06). However, that analysis was based on an earlier, largely pre-screening, period (1973–1996), and lacked information on adjuvant systemic therapy use. Previous studies examining cohorts of DCIS patients have reported a subsequent annual invasive CBC risk of 0.4–0.6%^13,15,16^, comparable to our finding.

When analyses were restricted to patients with information available on the mode of first BC detection, trends were similar overall. However, the higher CBC risk for DCIS patients compared with invasive BC was more pronounced within the not screen-detected BC group compared with the screen-detected BC group. Tumors not detected by screening could be interval tumors or those arising in women not attending for screening. Certainly, invasive interval tumors tend to be more aggressive than screen-detected BCs and hence receive more often adjuvant systemic treatment^22^.

We observed that the invasive CBCs developed within the DCIS group were less aggressive than the invasive CBCs developed after invasive first BC, i.e., more estrogen receptor positive (ER-positive), and lower tumor stage and grade. This may be explained by underlying etiological factors and/or be related to the use of adjuvant systemic therapy among invasive BC patients. Studies have shown that adjuvant systemic therapy influences subtype-specific CBC risk, e.g., endocrine therapy strongly reduces the risk of developing ER-positive CBC, but not ER-negative CBC^6,21^. This is supported by our subgroup analyses in patients with stage I BC not receiving adjuvant systemic therapy, who tended to develop similar CBC subtypes compared with DCIS patients.

The main strength of this study was the use of a large population-based nationwide cohort of DCIS and invasive BC patients, with complete follow-up on CBC over a long period. The NCR did not have follow-up information on distant metastases for all years included and therefore we could not take distant metastasis as a competing event into account. However, in the years where we had information on distant metastases (2003–2006), the median survival was 1.1 years and the 5-year overall survival after distant metastasis was fairly poor (6%). This indicates that death could be used as a proxy for distant metastasis. As we had complete information on death (as a competing event), we do not expect that the lack of information on distant metastases has led to an underestimation of the CBC risk. We also did not have information available about contralateral prophylactic mastectomy (CPM), which may have resulted in an underestimation of the CBC risk and may not have had equal uptake in all groups. According to Dutch guidelines^24^ only women carrying a *BRCA1 or BRCA2* germline mutation are advised to undergo a contralateral preventive mastectomy, as their CBC risk is high with an estimated 10-year risk of ~10–20%^25,26^ Unfortunately, information about *BRCA1* and *BRCA2* mutation was lacking. However, we do not expect that this missing information importantly influenced the results since only 1–2% of the DCIS population^27^, and 3–5% of the invasive BC population^25,28^ will be *BRCA1* or *BRCA2* mutation carriers. Finally, <1% of the DCIS patients were not treated according to the Dutch guideline since they received adjuvant chemotherapy, endocrine therapy, and/or trastuzumab. However, since this number is low, we do not expect that this affected our results.

Despite low CBC risks, the use of CPM has increased in recent years, both in patients diagnosed with invasive BC and in patients diagnosed with DCIS, especially in the United States^14,29^. Therefore, a need of individualized CBC risk prediction may be as important for patients diagnosed with DCIS as for patients with invasive BC. At present, CBC risk prediction models have been developed and validated for patients with invasive BC, but these models may not be appropriate for DCIS patients since most of the information available for invasive BC is not routinely collected in DCIS^18,19,30,31^. In our study, we had limited information on biological characteristics of DCIS, e.g., no information on receptor subtypes, and our multivariable model was therefore unable to differentiate CBC risk among DCIS patients. So, based on the clinical information currently available, CBC risk prediction in DCIS patients is insufficiently robust to be clinically actionable. More biological knowledge is needed to improve CBC prediction in DCIS patients.

Based on the results of this study we do not suggest to start treating DCIS patients with adjuvant systemic therapy to prevent CBC as the absolute invasive CBC risk is low. To facilitate patients and physicians in decision making, a comprehensive risk prediction model specifically developed for patients with DCIS would be desirable, including information on genetic, clinical, and lifestyle factors.

## Methods

### Study population

We evaluated 323,285 patients diagnosed with in situ or invasive first BC in 1989–2017, who underwent surgery, from the Netherlands Cancer Registry (NCR) (Supplementary Fig. 4). The NCR is an on-going nationwide population-based data registry of all newly diagnosed cancer patients in the Netherlands, with full coverage since 1989^32^. We excluded nine patients with first diagnosis without cytological or histological confirmation, 5785 with stage IV BC or with incomplete staging information, 66 with squamous cell carcinoma, and 4145 with in situ BC that was not pure DCIS (i.e., lobular, other subtype, or mixed with ductal). Follow-up for all patients started 3 months after the first diagnosis; therefore, 9,441 patients who had developed synchronous CBC (invasive or in situ), invasive ipsilateral BC, or died within 3 months after the first diagnosis were excluded.

### Patient and tumor characteristics

Clinico-pathological data were provided by the NCR. After notification by the nationwide network and registry of histo- and cytopathology in the Netherlands and the national hospital discharge database, registration clerks of the NCR collect data directly from patients’ records. Follow-up information on vital status and second cancers was complete up to 31 January 2018.

Staging was coded according to the TNM Classification of Malignant Tumors using the edition valid at the date of diagnosis, ranging from the 4th to the 8th edition^33^. If pathological stage was missing, clinical stage was used^34^.

Receptor status was determined by immunohistochemistry (IHC), and was included in the NCR since 2005. Tumors were defined as estrogen receptor (ER) positive or progesterone receptor (PR) positive when >10% of the tumor cells stained positive (from 2011 the threshold was ≥10%). A tumor was defined human epidermal growth factor receptor 2/neu-receptor (HER2) positive if IHC was 3+ (strong and complete membranous expression in >10% of tumor cells) or if IHC score 2+ when additional confirmation with in situ hybridization was available, but considered unknown if in situ hybridization confirmation was missing.

The NCR did not record information on *BRCA1 and BRCA2* germline mutation status and family history.

From 2011, the NCR recorded the mode of first BC detection, i.e., if the DCIS or invasive BC was screen-detected or not detected by screening. We did not have detailed information available on the tumors not detected by screening, but these may include interval tumors, non-screen attendant, or screened outside the national program (e.g., owing to family history). According to the Dutch guidelines, mammographic follow-up is similar for DCIS and invasive BC^24^.

Data used in this study were included in the NCR under an opt-out regime according to Dutch legislation and codes of conduct^34^. The NCR Privacy Review Board approved this study under reference number K18.245. Data were handled in accordance with privacy regulations for medical research^34^.

### Statistical analyses

The primary outcome was the development of metachronous CBC, defined as an invasive BC in the contralateral breast diagnosed at least three months after the first BC diagnosis (DCIS or invasive BC). Follow-up started three months after the first BC diagnosis, and ended at date of in situ- or invasive CBC, invasive ipsilateral BC, or last date of follow-up (owing to death, lost to follow-up, or end of study), whichever occurred first.

Cox proportional hazard models were performed to investigate the association of having DCIS compared with invasive BC as primary diagnosis with the cause-specific hazard of invasive CBC. We also performed analyses with in situ CBC, invasive ipsilateral BC, and death as the outcome. According to the Dutch guideline, DCIS patients do not receive adjuvant systemic therapy. We evaluated the impact of adjuvant systemic therapy by comparing the invasive CBC risk between DCIS patients and patients diagnosed with stage I BC not receiving adjuvant systemic therapy (no chemotherapy, endocrine therapy, nor trastuzumab), i.e., a subgroup of patients that resembles as much as possible the DCIS patient group in terms of treatment conditions. As hazard ratios (HRs) based on Cox regressions do not have a direct relationship with the cumulative incidence of the event of interest, we also performed competing risks regression to estimate the HRs for the subdistribution hazards of the Fine and Gray model^35,36^. In situ CBC, invasive ipsilateral BC, and death were considered as competing risks. We performed both univariable analyses and analyses adjusted for age- and year of first BC diagnosis. Since 1989, women in the Netherlands aged 50–70 have been invited for biannual screening by mammography, which was extended to women aged 75 since 1998. Based on this, we categorized age at first BC diagnosis into <50 years and ≥50 years. Based on the gradual implementation of the Dutch BC screening, we categorized year at first BC diagnosis into two periods: 1989–1998 (implementation phase) and 1999–2017 (full nationwide coverage; attendance rate is 78.8%^37^ and detection rate of invasive BC 6.6 per 1000 in 2017^38^ and for DCIS 0.94 per 1000 between 2004–2011^39^). We also performed our analyses stratified by mode of first BC detection. These analyses only included patients diagnosed during or after 2011 and aged 50–75 (eligible for screening).

Cumulative incidence curves of invasive CBC for DCIS patients, all invasive BC patients, and patients with stage I BC not receiving adjuvant systemic therapy were calculated considering in situ CBC, invasive ipsilateral BC, and death as competing risks. These curves were stratified by year of first BC diagnosis (1989–1998 and 1999–2017) and by age (<50 and ≥50 years).

We used joint Cox proportional hazard models^40^ to investigate subtype-specific CBC risk (according to stage, grade, ER, PR, and HER2 status) in DCIS patients compared with patients with invasive BC and compared with patients with stage I BC who did not receive adjuvant systemic therapy. Each model included subtype-specific CBC (e.g., ER-positive CBC, ER-negative CBC, ER unknown CBC), in situ CBC, ipsilateral invasive BC, and death as possible outcomes. As the NCR actively registered receptor status from 2005, these analyses only included patients diagnosed between 2005–2017.

Multivariable Cox regression was used to quantify the effect of clinico-pathological and treatment characteristics on CBC risk (all CBC and invasive CBC only) in DCIS patients. In addition, multivariable Fine and Gray regressions were performed to assess the association between every factor and the CBC cumulative incidence. Variables included in the models were age at first DCIS diagnosis, tumor grade, type of surgery (mastectomy or breast conserving surgery), and radiotherapy. The proportional hazard assumption of the models was assessed by examining the Schoenfeld residuals, and restricted cubic splines were used to verify whether linearity of age at first DCIS diagnosis would hold^41^. The discrimination ability of the models to identify patients developing CBC was calculated using the C-index^42^. Missing data were multiply imputed by chained equations to avoid loss of information owing to case-wise deletion causing bias and reduction in efficiency^43,44^. Multiple imputation accounts for missing data mechanisms assuming that the probability of missingness depends on the observed data namely missing at random. For every predictor with missing data, every imputation model selects predictors based on correlation structure underlying the data. Details about the imputation model are provided in Supplementary Methods.

Analyses were performed using STATA version 16.0, SAS (SAS Institute Inc., Cary, NC, USA) version 9.4, and R software version 3.5.3^45^.

### Reporting summary

Further information on research design is available in the Nature Research Reporting Summary linked to this article.

## Supplementary information

Supplementary Material

Reporting Summary Checklist FLAT

## Data Availability

The data sets generated and/or analyzed during the current study are not publicly available, as the study has used external data from the Netherlands Cancer Registry. The data sets will be made available from the Netherlands Cancer Registry upon reasonable request (data request study number K18.245). To apply for data access, please visit https://www.iknl.nl/en/ncr/apply-for-data. The data sets that support Figs. 1 and 2, and supplementary figs. 1–3, are publicly available in the figshare repository, in the following data record: 10.6084/m9.figshare.12982424^23^.

## Code availability

The codes developed during this study are available upon reasonable request. Analyses were performed using STATA version 16.0, SAS (SAS Institute Inc., Cary, NC, USA) version 9.4, and R software version 3.5.3.
