# Supplementary material for: Contralateral breast cancer risk in patients with ductal carcinoma in situ and invasive breast cancer
Source: NPJ Breast Cancer. 2020 Nov 3;6:60. doi: 10.1038/s41523-020-00202-8 (PMC7609533; doi:10.1038/s41523-020-00202-8)
Supplement: Supplementary file 1 — Supplementary Material [file 41523_2020_202_MOESM1_ESM.pdf]

Supplementary Table 1: Relative subsequent risks of death and invasive ipsilateral breast cancer after diagnosis with ductal carcinoma in situ versus invasive breast cancer using Cox and competing risk regression

Outcome(s)	Type of first BC	Cox regression		Competing risks regression	
		Unadjusted	Adjusted[a]	Unadjusted	Adjusted[a]
		HR (95% CI)	HR (95% CI)	HR[b] (95% CI)	HR[b] (95% CI)
Death	DCIS vs invasive BC	0.37 (0.36-0.38)	0.47 (0.45-0.49)	0.36 (0.35-0.37)	0.45 (0.44-0.47)
	DCIS vs stage I BC without adjuvant systemic therapy	0.56 (0.54-0.58)	0.71 (0.69-0.74)	0.53 (0.51-0.55)	0.68 (0.66-0.71)
Invasive IBC	DCIS vs invasive BC	6.67 (6.25-7.14)	6.68 (6.15-7.26)	7.69 (7.14-9.09)	7.79 (7.17-8.47)
	DCIS vs stage I BC without adjuvant systemic therapy	4.17 (3.85-4.54)	4.05 (3.68-4.45)	4.35 (4.00-4.76)	4.28 (3.90-4.71)

Abbreviations: HR: hazard ratio; CI: confidence interval; DCIS: ductal carcinoma in situ; BC: breast cancer; IBC: ipsilateral breast cancer

[a] Hazard ratios adjusted by age and year at first breast cancer diagnosis

[b] Hazard ratios for the subdistribution hazards of the Fine and Gray model. Invasive contralateral breast cancer, in situ contralateral breast cancer, invasive ipsilateral BC, and death were taken into account as competing risks

Supplementary Table 2: Cumulative incidence of invasive contralateral breast cancer at five and ten years in patients with ductal carcinoma in situ or invasive breast cancer by period and age at first diagnosis

	Period[a]	Type of first BC	Five-year cumulative incidence (%) (95% CI)	Ten-year cumulative incidence (%) (95% CI)
All	1989 - 1998	DCIS	2.2 (1.8 - 2.7)	4.4 (3.8 - 5.0)
		Invasive BC	2.5 (2.4 - 2.6)	4.5 (4.3 - 4.6)
		Stage I BC without adjuvant systemic therapy	2.9 (2.7 - 3.1)	5.5 (5.2 - 5.7)
	1999 - 2017	DCIS	2.5 (2.2 - 2.7)	5.0 (4.6 - 5.4)
		Invasive BC	1.9 (1.8 - 1.9)	3.8 (3.7 - 3.9)
		Stage I BC without adjuvant systemic therapy	3.0 (2.8 - 3.1)	5.8 (5.5 - 6.0)
Age < 50 years at first diagnosis	1989 - 1998	DCIS	2.3 (1.5 - 3.3)	4.6 (3.4 - 5.9)
		Invasive BC	3.2 (3.0 - 3.4)	5.5 (5.2 - 5.8)
		Stage I BC without adjuvant systemic therapy	3.4 (3.0 - 3.9)	6.1 (5.6 - 6.7)
	1999 - 2017	DCIS	2.4 (2.0 - 3.0)	4.7 (3.9 - 5.5)
		Invasive BC	1.7 (1.6 - 1.8)	3.5 (3.3 - 3.7)
		Stage I BC without adjuvant systemic therapy	2.9 (2.5 - 3.3)	5.5 (5.0 - 6.0)
Age ≥ 50 years at first diagnosis	1989 - 1998	DCIS	2.2 (1.8 - 2.7)	4.3 (3.7 - 5.0)
		Invasive BC	2.2 (2.1 - 2.3)	4.1 (4.0 - 4.3)
		Stage I BC without adjuvant systemic therapy	2.7 (2.5 - 2.9)	5.2 (4.9 - 5.5)
	1999 - 2017	DCIS	2.5 (2.2 - 2.7)	5.1 (4.7 - 5.4)
		Invasive BC	1.9 (1.8 - 2.0)	3.9 (3.8 - 4.0)
		Stage I BC without adjuvant systemic therapy	3.0 (2.8 - 3.1)	5.7 (5.6 - 6.0)

Abbreviations: CI: confidence interval; DCIS: ductal carcinoma in situ; BC: breast cancer
[a] The two periods were defined according to the gradual implementation of the screening program in the Netherlands: the implementation phase was between 1989 and 1998 and the full screening coverage was reached since 1999

Supplementary Table 3: Relative subsequent event risks after diagnosis with ductal carcinoma in situ versus invasive breast cancer by mode of first BC detection for patients diagnosed between 2011-2017[a]

		Overall		By mode of first BC detection[b]		
**Outcome**	**Type of first BC**	**Cox regression** HR (95% CI)[c]	**Competing risks regression** HR[c,d](95% CI)		**Cox regression** HR[c] (95% CI)	**Competing risks regression** HR[c,d] (95% CI)
**Death**	DCIS vs invasive BC (n=62,533, events=2,763)	0.48 (0.42-0.56)	0.48 (0.42-0.55)	screen-detected[e]	0.71 (0.60-0.83)	0.70 (0.60-0.83)
				not screen-detected[e]	0.33 (0.24-0.47)	0.33 (0.23-0.46)
	DCIS vs stage I BC without systemic therapy (n=27,288, events=701)	0.93 (0.79-1.09)	0.93 (0.79-1.09)	screen-detected[e]	1.04 (0.87-1.26)	1.05 (0.87-1.26)
				not screen-detected[e]	0.67 (0.46-0.98)	0.66 (0.45-0.97)
**Invasive IBC**	DCIS vs invasive BC (n=62,533, events=101)	5.12 (3.46-7.57)	5.17 (3.50-7.65)	screen-detected[e]	3.88 (2.46-6.14)	3.88 (2.46-6.14)
				not screen-detected[e]	10.19 (4.52-22.94)	10.42 (4.63-23.45)
	DCIS vs stage I BC without systemic therapy (n=27,288, events=83)	2.51 (1.62-3.91)	2.52 (1.62-.3.92)	screen-detected[e]	2.34 (1.41-3.88)	2.34 (1.41-3.88)
				not screen-detected[e]	3.46 (1.32-9.10)	3.48 (1.32-9.15)

Abbreviations: BC: breast cancer; HR: hazard ratio; CI: confidence interval; DCIS: ductal carcinoma in situ; IBC: ipsilateral breast cancer

[a] The analyses were performed in all patients diagnosed between 2011-2017, since from 2011 we had virtually complete information on the mode of first BC detection

[b] Results were based on interaction analyses including the interaction term between mode of first BC detection and type of first BC (type of first BC + mode of first BC detection + mode of first BC detection × type of first BC)

[c] Adjusted for age at first BC diagnosis

[d] Hazard ratios for the subdistribution hazards of the Fine and Gray model. Invasive CBC, in situ CBC, invasive ipsilateral BC, and death were taken into account as competing risks

[e] Not screen-detected includes interval tumours, non-screen attendant, or screened outside the national program

Supplementary Table 4: Joint Cox regression analyses assessing subtype-specific invasive contralateral breast cancer risk for patients with ductal carcinoma in situ compared to patients with invasive breast cancer[a]

	DCIS	All invasive BC	Stage I BC without adjuvant systemic therapy	DCIS vs Invasive BC	DCIS vs Stage I BC without adjuvant systemic therapy
**CBC subtypes**	N	N	N	HR (95%CI)	HR (95%CI)
**TNM stage**					
I	330	1,957	1,084	1.35 (1.20 - 1.52)	0.74 (0.65 - 0.83)
II	146	782	342	1.50 (1.26 - 1.79)	1.04 (0.86 - 1.26)
III	40	220	78	1.46 (1.04 - 2.05)	1.26 (0.86 - 1.86)
IV	8	143	29	0.45 (0.22 - 0.92)	0.72 (0.33 - 1.58)
**Tumor grade**					
I (well differentiated)	154	797	518	1.55 (1.31 - 1.84)	0.72 (0.60 - 0.86)
II (moderately differentiated)	245	1,253	652	1.57 (1.37 - 1.80)	0.91 (0.79 - 1.06)
III (poorly/undifferentiated)	95	675	251	1.13 (0.91 - 1.40)	0.93 (0.73 - 1.18)
**ER status**					
positive	386	2,081	1,151	1.49 (1.33 - 1.66)	0.81 (0.72 - 0.91)
negative	53	471	114	0.90 (0.69 - 1.19)	1.12 (0.81 - 1.56)
**PR status**					
positive	314	1,560	943	1.61 (1.43 - 1.82)	0.80 (0.71 - 0.91)
negative	119	971	311	0.98 (0.81 - 1.18)	0.93 (0.75 - 1.15)
**HER2 status**					
positive	51	250	91	1.63 (1.21 - 2.20)	1.35 (0.96 - 1.91)
negative	375	2,200	1,133	1.36 (1.22 - 1.52)	0.80 (0.71 - 0.90)

Abbreviations: CBC: contralateral breast cancer; DCIS: ductal carcinoma in situ; BC: breast cancer; HR: hazard ratio; CI: confidence interval; ER: estrogen receptor; PR: progesterone receptor; HER2: human epidermal growth factor receptor 2

[a] The analyses were performed only in patients diagnosed between 2005-2017, since from 2005 the Netherlands Cancer Registry actively registered receptor status

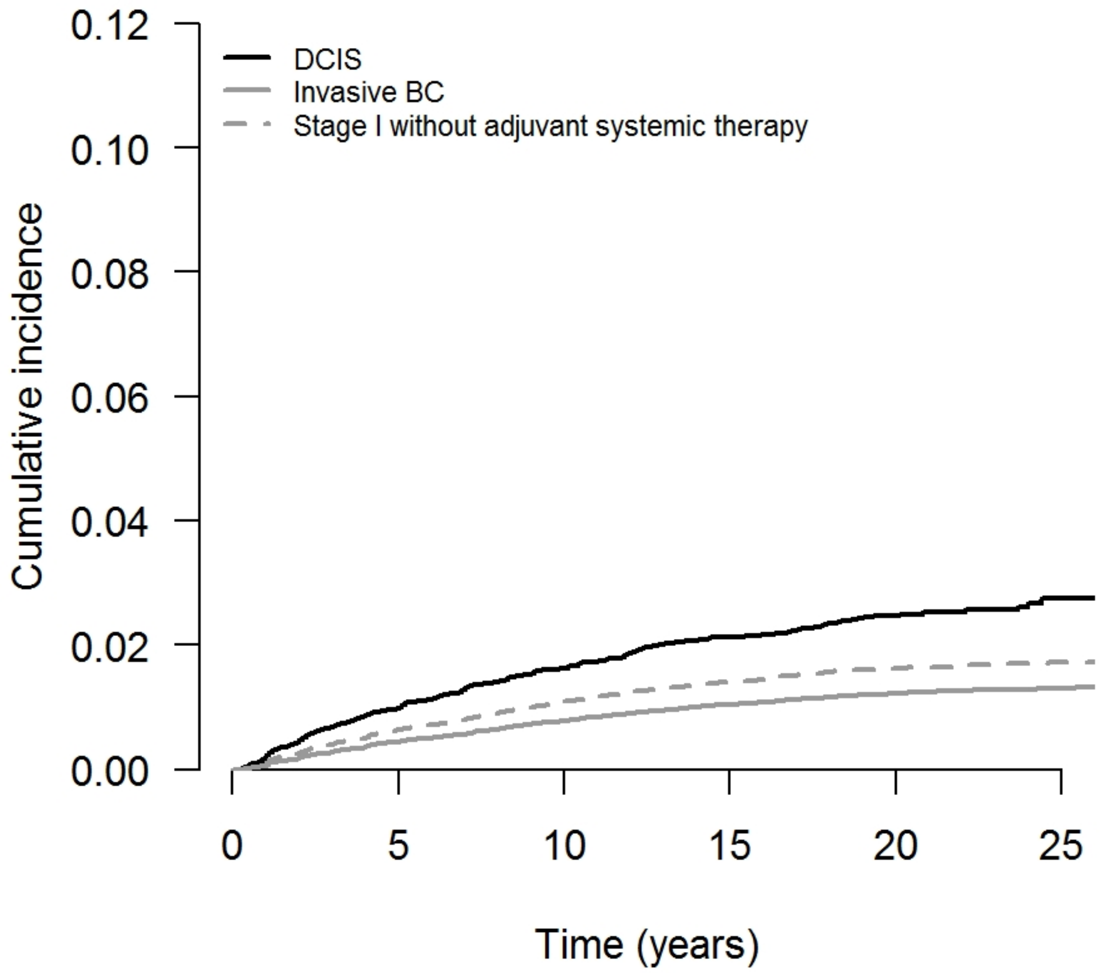

Supplementary Figure 1: Cumulative incidence of in situ contralateral breast cancer in patients diagnosed with ductal carcinoma in situ, invasive breast cancer (BC) stage I-III, and stage I breast cancer without (neo)adjuvant systemic therapy. The x-axis represents the time since the first breast cancer diagnosis (in years). The y-axis represents the cumulative incidence of in situ contralateral breast cancer. Abbreviations: DCIS: ductal carcinoma in situ; BC: breast cancer

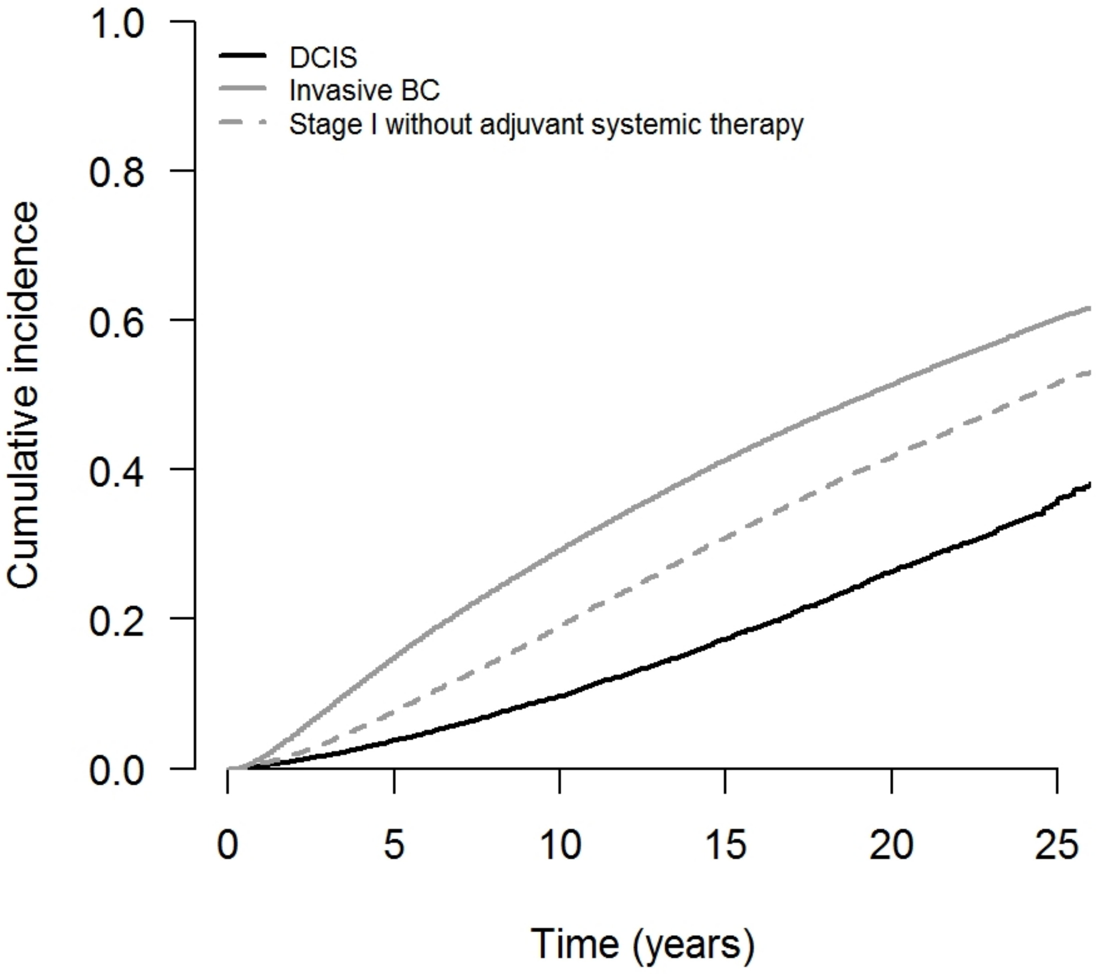

Supplementary Figure 2: Cumulative incidence of death in patients diagnosed with ductal carcinoma in situ, invasive breast cancer (BC) stage I-III, and stage I breast cancer without (neo)adjuvant systemic therapy. The x-axis represents the time since the first breast cancer diagnosis (in years). The y-axis represents the cumulative incidence of death. Abbreviations: DCIS: ductal carcinoma in situ; BC: breast cancer

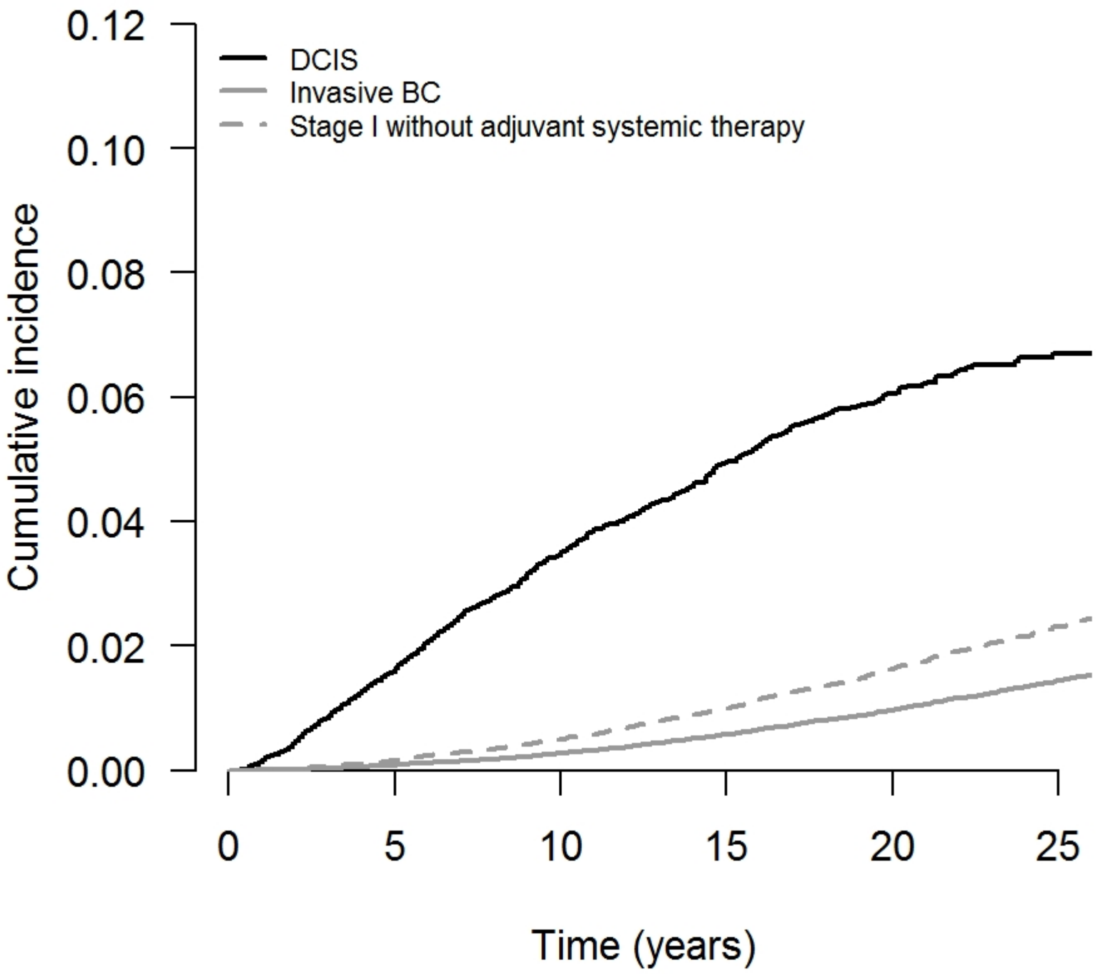

Supplementary Figure 3: Cumulative incidence of invasive ipsilateral breast cancer in patients diagnosed with ductal carcinoma in situ, invasive breast cancer (BC) stage I-III, and stage I breast cancer without (neo)adjuvant systemic therapy. The x-axis represents the time since the first breast cancer diagnosis (in years). The y-axis represents the cumulative incidence of invasive ipsilateral breast cancer. Abbreviations: DCIS: ductal carcinoma in situ; BC: breast cancer

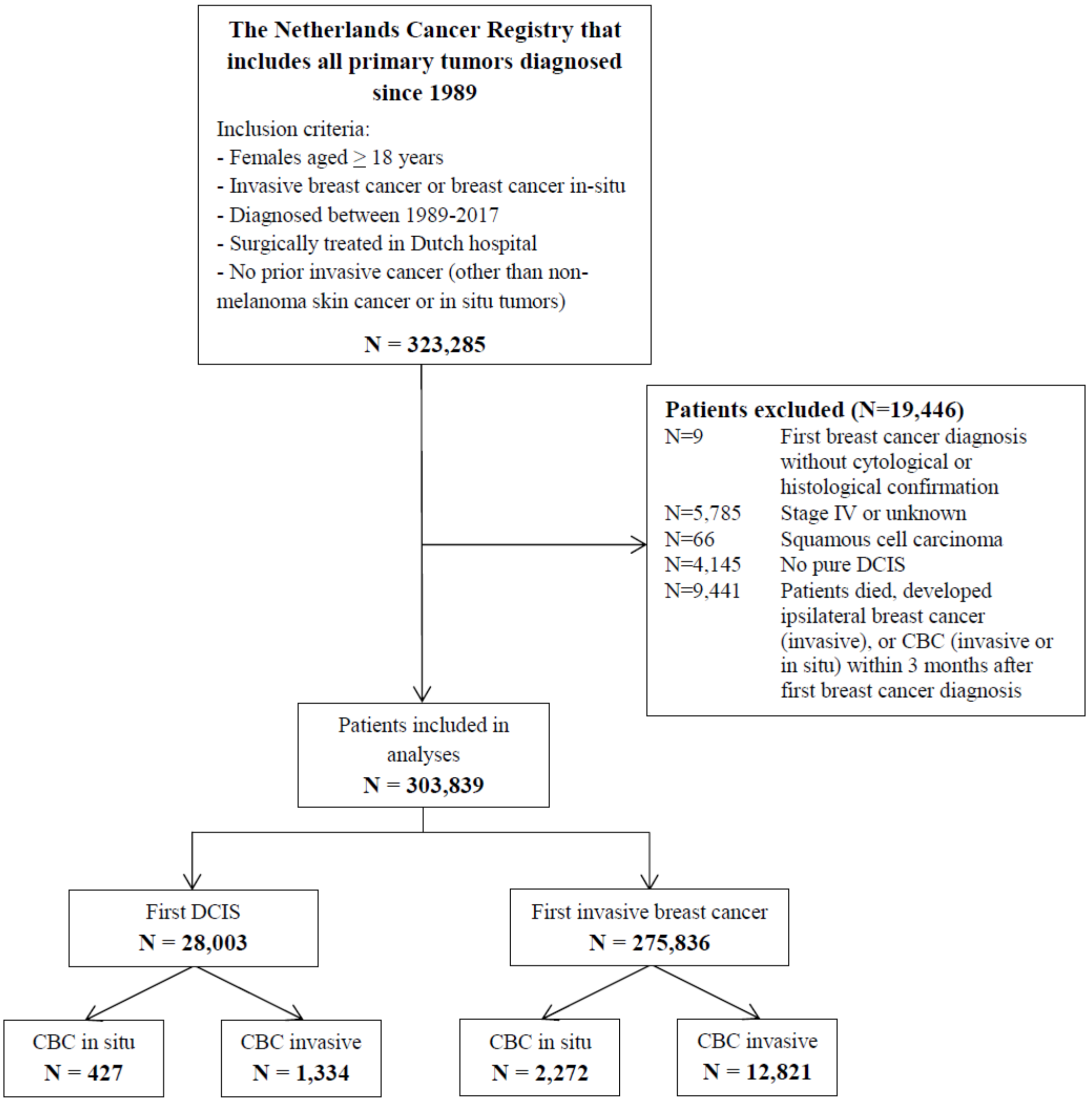

Supplementary Figure 4: Study flowchart. Abbreviations: DCIS: ductal carcinoma in situ; CBC: contralateral breast cancer

Supplementary Methods

Multiple imputation of missing values

The predictors for contralateral breast cancer with missing values among patients diagnosed with ductal carcinoma in situ (DCIS) were type of surgery to the breast (3.7%) and tumour grade (17.0%).

We used five imputed datasets based on the multiple imputation chained equations (MICE) using 50 iterations. The visit sequence of the variables was in ascending order of the number of missing values. This technique improves the accuracy and the statistical power assuming missing is at random (MAR)[1]. In the imputation procedure, we also used the year of DCIS diagnosis since this information provides a better correlation structure among covariates used as predictors in the imputation model. Continuous, binary and multiple categorical variables were imputed using predictive mean matching, binary and multinomial logistic regression, respectively. Time-to-event outcome defined as time to contralateral breast cancer, time to death, and time to ipsilateral breast cancer were included in the imputation process through the Nelson-Aalen cumulative hazard estimator[2]. For every variable with missing data, every imputation model selects predictors based on correlation structure underlying the data.

We used the R package mice (version 3.6.0) to impute our data and combine the estimates using Rubin’s rules.

Supplementary References

1. Van Buuren, S., *Flexible imputation of missing data*. Second ed. 2018: Chapman and Hall/CRC.
2. White, I.R. and P. Royston, *Imputing missing covariate values for the Cox model.* Stat Med, 2009. **28**(15): p. 1982-98.
